# A Hot Water Extract of *Curcuma longa* L. Improves Fasting Serum Glucose Levels in Participants with Low-Grade Inflammation: Reanalysis of Data from Two Randomized, Double-Blind, Placebo-Controlled Trials

**DOI:** 10.3390/nu14183763

**Published:** 2022-09-13

**Authors:** Ryusei Uchio, Chinatsu Okuda-Hanafusa, Ryosuke Saji, Kengo Kawasaki, Koutarou Muroyama, Shinji Murosaki, Yoshihiro Yamamoto, Yoshitaka Hirose

**Affiliations:** Research & Development Institute, House Wellness Foods Corp., 3-20 Imoji, Itami 664-0011, Japan

**Keywords:** turmeric (*Curcuma longa*), bisacurone, turmeronol, chronic inflammation, C-reactive protein (CRP), glucose

## Abstract

The dietary spice *Curcuma longa* L. (*C. longa*), also known as turmeric, has various biological effects. A hot water extract of *C. longa* was shown to have anti-inflammatory activities in preclinical and clinical studies. Chronic low-grade inflammation is associated with the disruption of glucose homeostasis, but the effect of *C. longa* extract on glucose metabolism in humans is poorly understood. Therefore, we investigated the effect of *C. longa* extracts on serum glucose levels in the presence of low-grade inflammation. We reanalyzed our published data from two randomized, double-blind, placebo-controlled trials in overweight participants aged 50 to 69 years and performed a stratified analysis using the inflammatory marker high-sensitivity C-reactive protein (hsCRP). In both studies, participants took a test food with a hot water extract of *C. longa* (*C. longa* extract group, *n* = 45 per study) or without *C. longa* extract (placebo group, *n* = 45 per study) daily for 12 weeks, and we measured the levels of serum hsCRP and fasting serum glucose. The mean baseline hsCRP value was used to stratify participants into two subgroups: a low-hsCRP subgroup (baseline mean hsCRP < 0.098 mg/dL) and a high-hsCRP subgroup (baseline mean hsCRP ≥ 0.098 mg/dL). In the low-hsCRP subgroup, we found no significant difference in fasting serum glucose levels between the two groups in either study, but in the high-hsCRP subgroup, the *C. longa* extract group had significantly lower levels of serum hsCRP (*p* < 0.05) and fasting serum glucose (*p* < 0.05) than the placebo group in both studies. In conclusion, a hot water extract of *C. longa* may help to improve systemic glucose metabolism in people with chronic low-grade inflammation.

## 1. Introduction

Glucose is a main source of cellular energy in mammalian cells and plays an important role in maintaining normal physiological function in humans [1]. Abnormally low levels of blood glucose can cause seizures, comas, and death. Islet tissue damage and various diseases such as type 2 diabetes cause elevated blood glucose levels [2]. Therefore, glucose homeostasis is tightly regulated through several processes, such as peripheral glucose uptake, gluconeogenesis, glycolysis, glycogenesis, and glycogenolysis [1]. These processes are controlled by glucoregulatory hormones, such as insulin, incretin, glucagon, glucocorticoid, and growth hormones released from some organs, including the pancreas, intestine, adrenal gland, and pituitary gland [3,4]. However, various factors related to aging, obesity, and an unhealthy lifestyle are known to induce pancreatic β-cell dysfunction, insulin resistance, and the disruption of systemic glucose homeostasis, resulting in the progression of type 2 diabetes [5,6].

Chronic low-grade inflammation is an ongoing inflammatory response. This type of inflammation is mediated by a long-lasting immune response and can cause tissue damage, inhibit wound healing, and impair glucose homeostasis [5,6]. A standard definition of chronic low-grade inflammation has not yet been determined, but various causes have been identified, including aging [7], obesity [8], and an unhealthy lifestyle [9] without any apparent infection or tissue injury [10]. These play a key role in the development of pancreatic β-cell dysfunction, insulin resistance, and type 2 diabetes. Anti-inflammatory drugs have been shown to improve insulin secretion, insulin resistance, and blood glucose levels in clinical intervention studies [11,12]. Recent research revealed that serum levels of high-sensitivity C-reactive protein (hsCRP) are only slightly elevated in chronic low-grade inflammation compared with acute inflammation [8,10]. However, previous reports indicated that participants with mildly elevated hsCRP may have a higher risk of impaired fasting glucose and type 2 diabetes [13,14].

The medicinal herb *Curcuma longa* L. (*C. longa*), also known as turmeric, is a member of the Zingiberaceae family and has various physiological activities [15]. Water extracts of *C. longa* have antioxidant and anti-inflammatory effects [16,17,18,19]; they can prevent skeletal muscle atrophy [20], improve the water content of skin [21], promote corneal wound healing [22], and show positive effects on fatigue [23]. In addition, in animal models, water extracts of *C. longa* were reported to prevent various chronic inflammatory diseases, including cotton pellet-induced granuloma [24], carbon tetrachloride-induced hepatitis [25], and non-alcoholic steatohepatitis [26], by reducing the production of inflammatory cytokines, chemokines, and cell adhesion molecules. Furthermore, previous intervention studies showed that daily intake of a hot water extract of *C. longa* improved levels of systemic inflammatory markers such as hsCRP, tumor necrosis factor (TNF)-α, and interleukin (IL)-6 [27,28]. *C. longa* extract was recently shown to decrease blood glucose levels in an animal model of dietary-induced obesity and streptozotocin-induced diabetes [29,30]. However, the influence of water extract of *C. longa* on glucose metabolism in humans is not clearly understood.

Therefore, we reanalyzed our data from two randomized, double-blind, placebo-controlled trials to investigate the effects of *C. longa* extract on serum glucose levels in humans with low-grade inflammation. We then reanalyzed our data [27,28] and performed a stratified analysis by using the inflammatory marker hsCRP.

## 2. Materials and Methods

### 2.1. Study Design

**Figure 1:**
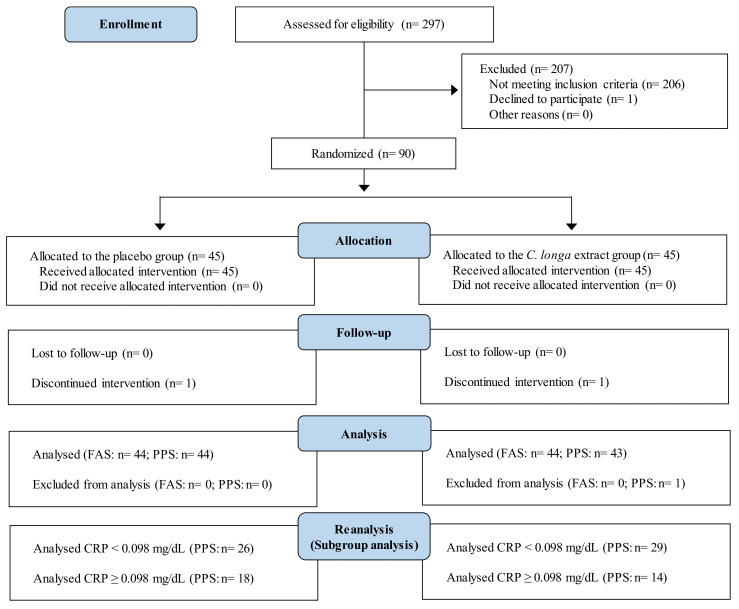
Study flow diagram (CONSORT 2010) in study 1.

**Figure 2:**
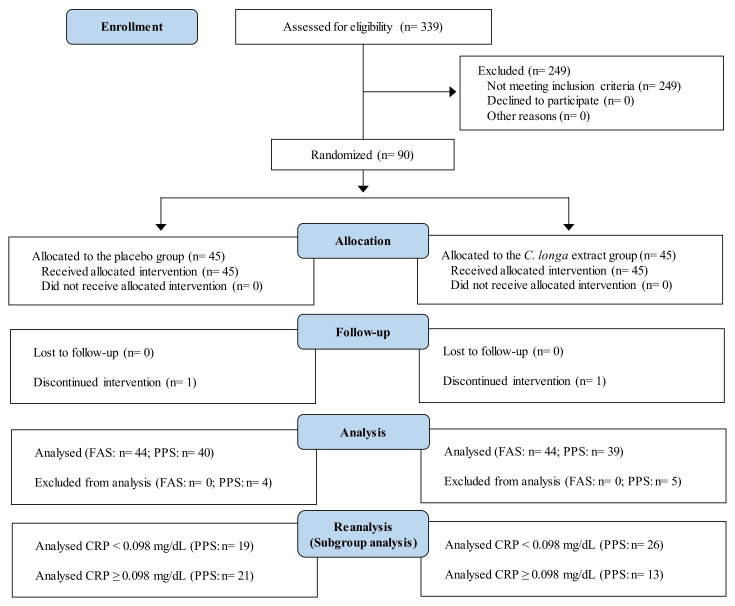
Study flow diagram (CONSORT 2010) in study 2.

We reanalyzed data from two randomized, double-blind, placebo-controlled trials (*Nutrients*, 11 (8): 1822, 2019 [study 1; [27]] and *Nutr J*, 20 (1): 91, 2021 [study 2; [28]]). All procedures in the two studies involving human participants were approved by the institutional review board of Chiyoda Paramedical Care Clinic (Tokyo, Japan). The studies were conducted according to the Declaration of Helsinki. Before enrollment in the study, all participants provided written informed consent. Both studies were performed by a contract research organization (CRO; CPCC Co., Ltd., Tokyo, Japan) at the Chiyoda Paramedical Care Clinic; study 1 was performed from June to December 2017 (University Hospital Medical Information Network registration number UMIN000029095), and study 2 was performed from June to December 2019 (UMIN000037370). Figure 1 and Figure 2 show the Consolidated Standards of Reporting Trials (CONSORT) 2010 diagrams of the flow of participants from enrollment to analysis in studies 1 and 2, respectively; the completed CONSORT checklists are provided in the original research papers [27,28].

### 2.2. Enrolment of Participants

#### 2.2.1. Study 1

Among people attending the Chiyoda Paramedical Care Clinic, 297 potential participants were consecutively assessed for eligibility. The study included men and menopausal women aged 50 to 69 years with a body mass index (BMI) between overweight and obesity class 1 (≥23 to <30 kg/m^2^) [31] or blood pressure between prehypertension and grade 1 (mild) hypertension (systolic blood pressure ≥120 to <160 mmHg or diastolic blood pressure ≥80 to <100 mmHg). The exclusion criteria were as follows: (1) positive for hepatitis C virus antibody or hepatitis B surface antigen, (2) use of medications or health foods that could possibly influence the results of this study, (3) history of heart, liver, kidney, or gastrointestinal disease, (4) history of cardiovascular disease, (5) excessive alcohol intake (mean daily consumption of 60 g or more) or smoking (mean daily consumption of two packs), (6) extremely irregular dietary habits, (7) allergies to medications or foods, (8) participation in another trial, either currently or in the past 4 weeks, or a plan to participate in another trial during the scheduled study period, (9) donation of blood within 1 month before the study, (10) in men, donation of 400 mL of blood within 3 months before the study, (11) in women, donation of 400 mL of blood within 4 months before the study, (12) in men, donation of more than 1200 mL of blood minus the estimated volume of blood collected during the study within 1 year before the study, (13) in women, donation of more than 800 mL of blood minus the estimated volume of blood collected during the study within 1 year before the study, and (14) judged to be unsuitable for the study for other reasons by the investigators.

#### 2.2.2. Study 2

Among people attending the Chiyoda Paramedical Care Clinic, 339 potential participants were consecutively assessed for eligibility. The study included overweight men and menopausal women aged 50 to 69 years (BMI ≥ 23 to <30 kg/m^2^) [31], with blood glucose less than 126 mg/dL and high-density blood cholesterol greater than or equal to 35 mg/dL. The exclusion criteria were as follows: (1) positive for hepatitis C virus antibody or hepatitis B surface antigen, (2) use of medications or health foods that could possibly influence the results of this study, (3) history of heart, liver, kidney, or gastrointestinal disease, (4) history of circulatory disease, (5) excessive alcohol intake (mean daily consumption of 60 g or more), (6) excessive smoking (mean daily consumption of two packs or more), (7) extremely irregular dietary habits, (8) allergies to medications or foods (especially soybeans or gelatin), (9) participation in another trial, either currently or in the past 4 weeks, or a plan to participate in another trial during the scheduled study period, (10) donation of blood within 1 month before the study, (11) in men, donation of 400 mL of blood within 3 months before the study, (12) in women, donation of 400 mL of blood within 4 months before the study, (13) in men, donation of more than 1200 mL of blood minus the estimated volume of blood collected during the study within 1 year before the study, (14) in women, donation of more than 800 mL of blood minus the estimated volume of blood collected during the study within 1 year before the study, and (15) judged to be unsuitable for the study for other reasons by the investigators.

### 2.3. Study Agent

The composition of the test diets used in the two studies is shown in Table 1. The turmeric diets contained a hot water extract of *C. longa* (House Wellness Foods) and were prepared according to the method previously described in [27,28]. The placebo diets contained coloring agents to match the turmeric diet’s color.

### 2.4. Intervention

The intervention was conducted according to the procedures described in the publications of the two studies [27,28]. Briefly, the CRO selected 90 participants who satisfied the inclusion and exclusion criteria for the respective study (Figure 1 and Figure 2). The test diets and the selected participants were randomly assigned numbers, and the assignment list was stored carefully until the database was locked. Throughout the study, all participants and investigators were blind to the treatment provided. The participants were randomly allocated into two groups by stratified randomization. Each participant consumed a test diet with *C. longa* extract (*C. longa* extract group, *n* = 45) or without (placebo group, *n* = 45) once daily for 12 weeks. Participants visited the study center during weeks 0, 4, 8, and 12 to undergo an interview conducted by an experienced physician, physiological measurements, hematology and biochemistry tests, and urinalysis, and to complete questionnaires. The hematology and biochemistry tests and urinalysis were performed by a contracted laboratory company (LSI Medience Co., Ltd., Tokyo, Japan). The participants were asked to daily record the following information in a diary until the end of the study: occurrence of diseases and symptoms, intake of the study diet, healthy foods, medications, reasons for taking medications, the dosage, and the duration of use.

### 2.5. Measurement of Serum hsCRP Level

The serum levels of hsCRP were measured by an immunonephelometric method with an upper detection limit of 0.500 mg/dL (LSI Medience) [32]. For samples exceeding this limit, a remeasurement was performed by standard latex agglutination turbidimetry using N-assay LA CRP-T kits (Nittobo Medical Co., Ltd., Tokyo, Japan) [33].

### 2.6. Measurement of Fasting Serum Glucose Level

The level of fasting serum glucose was measured by commercial glucose assay kits based on the glucokinase method (LSI Medience) [32,34].

### 2.7. Sample Size

To calculate the minimum number of participants required for adequate statistical power, we used the G Power 3.1.9 program (University of Düsseldorf, Düsseldorf, Germany). In a previous clinical study, a green tea extract with anti-inflammatory activity reduced the serum CRP level by about 30% from the baseline [35]. Therefore, a sample size of 45 participants per group was estimated to be sufficient for the present study based on the following assumptions: a 30% reduction of serum CRP by *C. longa* extract, Cohen’s *d*-value of 0.60, a statistical power of 80%, and type I error of 5% (two-tailed).

### 2.8. Stratified Analysis

To investigate the effect of a *C. longa* extract on serum glucose levels in the presence of low-grade inflammation, we stratified the participants according to the baseline mean value of hsCRP (0.098 mg/dL) in study 1 (Table S1) and divided them into two subgroups: a low-hsCRP subgroup (baseline mean hsCRP < 0.098 mg/dL) and a high-hsCRP subgroup (baseline mean hsCRP ≥ 0.098 mg/dL).

### 2.9. Statistical Analysis

Statistical analysis was performed in the intention-to-treat (ITT) population, which was defined as all randomized participants. The full analysis set (FAS) was used to assess safety, and the per-protocol set (PPS) was used to evaluate efficacy. In the efficacy assessment of inflammatory markers, we excluded the data of participants who were suspected of having acute inflammation because of a markedly increasing hsCRP level or symptoms associated with acute inflammation as diagnosed by a physician. All statistical analyses were performed with the IBM SPSS statistical software package (version 26) for Windows (IBM Corp., Armonk, NY, USA). Results are presented as the mean (standard deviation [SD]). Baseline characteristics were compared between the two groups by the two-tailed unpaired Student’s *t*-test when variance was homogeneous or the Aspin-Welch *t*-test when variance was heterogeneous, except for sex-related results, which were analyzed by the two-tailed Mann-Whitney *U*-test. Changes from the baseline were analyzed by repeated measures of two-way analysis of variance (ANOVA; two groups × three-time points) with the SPSS general linear model for determining the main effects of the group and time and their interaction, followed by a comparison between the *C. longa* extract and placebo groups at each time point with simple tests for main effect [27,36,37,38]. A probability (*p*) value less than 0.05 was considered to indicate statistical significance.

## 3. Results

### 3.1. Subjects

#### 3.1.1. Study 1

The flow of participants through the study is shown in Figure 1. The 90 participants were randomly allocated to the *C. longa* extract or placebo group (*n* = 45 per group), and 87 participants completed the study: two participants dropped out before completing the study because of cerebral infarction (*n* = 1 in the placebo group) and ovarian cystectomy (*n* = 1 in the *C. longa* extract group); one participant did not consume the test agent in compliance with the protocol (*n* = 1 in the *C. longa* extract group) and was therefore excluded from the efficacy analysis (PPS analysis). The baseline characteristics showed no significant differences between the two groups (Table S1). The mean intake of the test diets also showed no significant differences between the two groups. In the stratified analysis, the baseline characteristics showed no significant differences between the *C. longa* extract and placebo groups in either the low- or high-hsCRP subgroup (Table 2).

#### 3.1.2. Study 2

The flow of participants through the study is shown in Figure 2. The 90 participants were randomly allocated to the *C. longa* extract group or the placebo group (*n* = 45 per group), and 79 participants completed the study: two participants dropped out before completing the study because they declined to continue participation because of a marked reduction of body weight (*n* = 1 in the placebo group) and a bone fracture resulting from a slip-and-fall accident while walking (*n* = 1 in the *C. longa* extract group); nine participants were excluded from the efficacy assessment (PPS analysis) because they did not comply with the protocol, i.e., they changed their dietary habits (*n* = 4 in the *C. longa* extract group and *n* = 4 in the placebo group) or fractured a bone as a result of a slip-and-fall accident while cycling (*n* = 1 in the *C. longa* extract group). Baseline characteristics showed no significant differences between the *C. longa* extract and placebo groups, except for significantly higher serum glucose levels in the placebo group compared with the *C. longa* extract group (Table S2). The mean intake of the test diets showed no significant differences between the two groups. In the stratified analysis, baseline characteristics showed no significant differences between the *C. longa* extract and placebo groups in the low-hsCRP subgroup, but in the high-hsCRP subgroup, LDL cholesterol was significantly lower at baseline in the *C. longa* extract group than in the placebo group (Table 3).

### 3.2. Effect of C. longa Extract on Serum hsCRP Levels

#### 3.2.1. Study 1

In the low-hsCRP subgroup, the change of hsCRP from baseline was significantly lower in the *C. longa* extract group than in the placebo group throughout the study period (*p* = 0.013 by r-ANOVA); in addition, the hsCRP level tended to be lower in week 8 (*p* = 0.084) and significantly lower in week 12 (*p* = 0.006) in the *C. longa* extract group than in the placebo group (Table 4). In the high-hsCRP subgroup, the change of hsCRP from baseline showed no significant differences between the two groups throughout the study period (r-ANOVA); however, the hsCRP level was significantly lower in week 8 (*p* = 0.007) and tended to be lower in week 12 (*p* = 0.095) in the *C. longa* extract group than in the placebo group (Table 4).

#### 3.2.2. Study 2

In the low-hsCRP subgroup, the change of hsCRP from baseline did not show any significant differences between the two groups (Table 5). In the high-hsCRP subgroup, the change of hsCRP from baseline was significantly lower in the *C. longa* extract group than in the placebo group (*p* = 0.001 by r-ANOVA). Furthermore, in the high-hsCRP subgroup, the hsCRP level tended to be lower in week 4 (*p* = 0.076) and significantly lower in week 8 (*p* = 0.037) and week 12 (*p* = 0.014) in the *C. longa* extract group than in the placebo group (Table 5).

### 3.3. Effect of C. longa Extract on Fasting Serum Glucose Levels

#### 3.3.1. Study 1

In the low-hsCRP subgroup, the change in glucose from baseline did not show any significant differences between the two groups (Table 4). In the high-hsCRP subgroup, the change of glucose from baseline tended to be lower in the *C. longa* extract group than in the placebo group throughout the study period (*p* = 0.052 by r-ANOVA), and the serum glucose level was significantly lower in week 4 (*p* < 0.001), week 8 (*p* = 0.006), and week 12 (*p* < 0.001) in the *C. longa* extract group than in the placebo group (Table 4).

#### 3.3.2. Study 2

In the low-hsCRP subgroup, the change of glucose from baseline showed no significant differences between the two groups throughout the study period (r-ANOVA), but the serum glucose level was significantly higher in week 4 (*p* = 0.003) in the *C. longa* extract group than in the placebo group (Table 5). In the high-hsCRP subgroup, the change of glucose from baseline showed no significant differences between the two groups throughout the study period (r-ANOVA), but the serum glucose level was significantly lower in week 12 (*p* = 0.022) in the *C. longa* extract group than in the placebo group (Table 5).

### 3.4. Safety of the Intervention

Adverse events were assessed in the ITT population (*C. longa* extract group, *n* = 45; placebo group, *n* = 45), and safety parameters were assessed in the FAS population (Figure 1 and Figure 2). These results were described in detail in the original research papers [27,28]. In brief, some adverse events were observed in both studies, but they were mild, and an experienced physician judged that they were unrelated to the dietary intervention. In study 1, the male participants showed elevated levels of aspartate aminotransferase and creatinine above the corresponding reference ranges, but the results of the other hematology and biochemistry tests, urinalysis, and physiological tests did not differ significantly between the two groups. In study 2, the safety parameters did not show any significant differences between the two groups.

## 4. Discussion

To investigate the effects of a hot water extract of *C. longa* on fasting serum glucose levels and assess the influence of low-grade inflammation, we reanalyzed our published data from two randomized, double-blind, placebo-controlled trials in overweight participants aged 50 to 69 [27,28]. We performed a stratified analysis by dividing participants into two groups based on baseline serum hsCRP level. In the low-hsCRP subgroup, we found no significant differences in serum glucose levels between the *C. longa* extract and placebo groups, but in the high-hsCRP subgroup, we found that intake of the *C. longa* extracts significantly improved levels of serum hsCRP and fasting serum glucose. These results suggest that intake of *C. longa* extract could potentially improve systemic glucose metabolism in people with low-grade inflammation.

Glucose metabolism is tightly regulated by various hormones, such as insulin and glucagon [1,3]. However, chronic low-grade inflammation is known to induce β-cell dysfunction and insulin resistance and disrupt glucose homeostasis [6]. The standard test for CRP cannot detect the small increases in CRP levels seen in low-grade inflammation, but the hsCRP assay can be used as a marker of such inflammation [8,10]. Epidemiological studies have reported that participants with a slight elevation of hsCRP levels (≥0.10 mg/dL) have a potential risk of impaired fasting glucose (a marker of prediabetes) and type 2 diabetes [13,14]. In previous large-scale observational studies, the risk of type 2 diabetes has been shown to be lower in people with anti-inflammatory agents such as aspirin than in people without these agents [39,40]. In the present study, the *C. longa* extract significantly improved the levels of serum hsCRP and fasting serum glucose in participants with mildly elevated hsCRP (≥0.098 mg/dL) in both clinical studies (Table 4 and Table 5). Therefore, these results suggest that *C. longa* extract may improve systemic glucose metabolism associated with chronic low-grade inflammation and could possibly decrease the risk of prediabetes and type 2 diabetes.

TNF-α and IL-1β are pro-inflammatory cytokines and are known to have negative effects on the action of insulin, such as impairing glucose uptake by inhibiting the phosphatidylinositol-3-kinase (PI3K) and protein kinase B (Akt) pathway and promoting the de-elopement of insulin resistance [41]. The transcription nuclear factor kappa B (NF-kB) is known to be activated by various inflammatory stimuli and induce the expression of inflammatory-associated genes, including TNF-α and IL-1β. Inhibition of NF-kB activation improved insulin resistance in leptin-deficient obese mouse models [42]. In addition, anti-inflammatory drugs targeting TNF-α, IL-1β, and NF-kB improved insulin resistance and fasting blood glucose levels in clinical studies [11,12]. In a study in a non-alcoholic steatohepatitis mouse model, the hot water extract of *C. longa* inhibited the hepatic production of TNF-α, IL-1β, and IL-6 [26], and in our earlier clinical studies, *C. longa* extract improved systemic inflammatory markers, including hsCRP, TNF-α, and IL-6 [27,28]. Some components in aqueous extracts of *C. longa* have anti-inflammatory activity. For example, turmeronol A and B derived from a water extract of *C. longa* were reported to inhibit the production of TNF-α, IL-1β, and IL-6 and the activation of NF-kB in activated macrophages [43]. Bisacurone, another component of *C. longa* extracts, also suppressed the NF-kB signaling pathway by inhibiting phosphorylation of NF-kB inhibitor protein alpha (IkBα) and nuclear translocation of NF-kB in endothelial cells stimulated with TNF-α [44]. Our results suggest that *C. longa* extract may improve systemic glucose levels. We hypothesize that it does so by suppressing the activation of the NF-kB signaling pathway and the production of inflammatory cytokines.

Peripheral tissues such as the pancreas, skeletal muscle, and liver are important organs for regulating systemic glucose homeostasis [3,45]. In response to hyperglycemia, pancreatic β-cells secrete insulin, which binds to its receptor, activates PI3/Akt signaling, induces the translocation of glucose transporter 4 to the plasma membrane, and promotes glucose uptake in various cells, such as skeletal muscle cells [3,45]. Hepatic glucose production contributes to regulating glucose homeostasis, and insulin decreases the systemic glucose level by inhibiting the production of glucose by the liver [46]. However, chronic inflammation is known to induce pancreatic β-cell injury and dysfunction, inhibit insulin-mediated glucose uptake and lead to hepatic insulin resistance, as characterized by impaired insulin-induced suppression of hepatic gluconeogenesis [8,46]. An aqueous extract of *C. longa* was reported to decrease lipid peroxidation-induced DNA damage [47], and an herb mixture containing *C. longa* extract was shown to protect pancreatic β-cell injury from an inflammatory cytokine cocktail consisting of TNF-α, IL-1β, and interferon-γ [48]. In addition, an ex vivo study found that an aqueous extract of *C. longa* increased the secretion of insulin in pancreatic tissue and promoted insulin-induced glucose uptake in muscle tissue by activating PI3K/Akt signaling [49,50]. Bisacurone also was shown to induce hepatic activation of AMP-activated kinase (AMPK) and peroxisome proliferator-activated receptor (PPAR)-α proteins in in vitro and in vivo studies [51]. Furthermore, in animal models, the administration of fenofibrate or aminoimidazole-4-carboxamide riboside (AICAR), activators of PPAR-α and AMPK, respectively, improved hepatic insulin resistance [52,53]. Thus, *C. longa* extract may reduce cell injury and dysfunction in pancreatic β-cells and improve glucose uptake and insulin resistance in peripheral tissues, resulting in the improvement of systemic glucose levels.

## 5. Conclusions

To investigate the effects a hot water extract of *C. longa* on fasting serum glucose levels and assess the influence of low-grade inflammation, we reanalyzed data from two randomized, double-blind, placebo-controlled trials in overweight participants aged 50 to 69 and performed a stratified analysis by dividing participants into two subgroups based on baseline hsCRP levels. In the low-hsCRP subgroup, we found no significant differences in the serum glucose level between the two groups. In contrast, in the high-hsCRP subgroup, the *C. longa* extract group showed significantly lower levels of serum hsCRP and fasting serum glucose than the placebo group. These results suggest that daily intake of a hot water extract of *C. longa* may have the potential to improve systemic glucose metabolism in people with chronic low-grade inflammation.

## Figures and Tables

**Table 1:** Composition of test diets.

	Study 1		Study 2	
	Placebo (0.90 g/3 Tablets)	*C. longa* Extract (0.90 g/3 Tablets)	Placebo (0.97 g/2 Capsules)	*C. longa* Extract (0.97 g/2 Capsules)
Energy, Kcal	3.4	3.4	6.1	5.5
Carbohydrate, g	0.82	0.76	0.24	0.23
Protein, g	0	0.02	0.20	0.29
Lipid, g	0.01	0.03	0.47	0.38
Sodium chloride, mg	0.74	0.45	0.66	0.13
Bisacurone, μg	0	400	0	400
Turmeronol A, μg	0	80	0	100
Turmeronol B, μg	0	20	0	100

**Table 2:** Baseline characteristics of the participants stratified by serum hsCRP levels in study 1 ^1^.

	Low-hsCRP Subgroup (<0.098 mg/dL)				High-hsCRP Subgroup (≥0.098 mg/dL)			
	Placebo (*n* = 26)		*C. longa* Extract (*n* = 29)		Placebo (*n* = 18)		*C. longa* Extract (*n* = 14)	
	Mean	SD	Mean	SD	Mean	SD	Mean	SD
Sex, male/female, *n*	16/10		17/12		6/12		6/8	
Age, y	57.8	5.4	59.0	5.5	59.6	5.6	58.3	5.1
Physical measurements and tests								
Height, cm	163.8	9.1	163.1	6.8	159.7	5.8	162.2	11.0
Body weight, kg	67.4	10.8	65.4	7.3	65.0	6.9	68.7	8.6
BMI, kg/m^2^	25.1	2.9	24.5	1.5	25.4	1.9	26.1	2.0
SBP, mmHg	134.6	18.3	128.6	14.3	125.7	13.2	130.6	18.0
DBP, mmHg	84.1	12.4	80.5	10.4	80.1	10.3	83.0	10.5
Serum inflammatory markers								
hsCRP, mg/dL	0.055	0.019	0.059	0.026	0.182	0.079	0.165	0.068
Metabolic markers								
Glucose, mg/dL	83.2	5.8	85.8	4.7	85.7	6.2	86.6	10.4
HbA1c, %	5.39	0.18	5.50	0.27	5.60	0.26	5.52	0.30
Triglyceride, mg/dL	106.8	48.1	118.6	79.5	120.7	66.9	148.2	62.0
Total cholesterol, mg/dL	214.7	37.1	210.1	33.2	228.0	40.4	234.1	30.0
LDL-cholesterol, mg/dL	133.0	30.8	126.1	30.1	145.4	38.2	144.4	26.0
HDL-cholesterol, mg/dL	55.5	18.1	55.7	12.5	52.6	8.7	56.5	12.2

BMI, body mass index; *C. longa*, *Curcuma longa* L.; DBP, diastolic blood pressure; HbA1c, hemoglobin A1c; HDL, high-density lipoprotein; hsCRP, high-sensitivity C-reactive protein; LDL, low-density lipoprotein; SBP, systolic blood pressure. ^1^ Values represent the means and standard deviations for *n* = 26 (placebo group) or *n* = 29 (*C. longa* extract group) in the participants with a low-hsCRP level (<0.098 mg/dL). *n* = 18 (placebo group) or *n* = 14 (*C. longa* extract group) in the participants with a high-hsCRP level (≥0.098 mg/dL). Results in men and women were compared with the two-tailed Mann Whitney *U*-test, and physical measurements and tests, serum inflammatory markers, and metabolic markers were compared with the two-tailed unpaired Student’s *t*-test when variance was homogeneous or the Aspin-Welch *t*-test when variance was heterogeneous.

**Table 3:** Baseline characteristics of the participants stratified by serum hsCRP protein levels in study 2 ^1^.

	Low-hsCRP Subgroup (<0.098 mg/dL)				High-hsCRP Subgroup (≥0.098 mg/dL)			
	Placebo (*n* = 19)		*C. longa* Extract (*n* = 26)		Placebo (*n* = 21)		*C. longa* Extract (*n* = 13)	
	Mean	SD	Mean	SD	Mean	SD	Mean	SD
Sex, male/female, *n*	8/11		11/15		8/13		4/9	
Age, y	56.5	4.4	56.1	4.6	56.7	4.4	57.9	5.7
Physical measurements and tests								
Height, cm	164.4	8.7	162.8	7.8	160.8	8.1	161.9	10.1
Body weight, kg	70.5	7.7	71.0	6.9	69.4	8.4	68.9	9.5
BMI, kg/m^2^	26.1	1.8	26.8	1.5	26.8	1.7	26.2	1.6
SBP, mmHg	120.8	14.0	121.3	14.1	124.8	11.6	116.5	16.1
DBP, mmHg	76.9	11.6	78.3	9.4	78.2	9.0	76.2	10.1
Serum inflammatory markers								
hsCRP, mg/dL	0.042	0.025	0.049	0.031	0.126	0.016	0.142	0.078
Metabolic markers								
Glucose, mg/dL	93.5	10.8	88.0	6.9	91.1	6.3	89.5	5.5
HbA1c, %	5.63	0.22	5.58	0.26	5.63	0.19	5.48	0.26
Triglyceride, mg/dL	125.2	61.0	128.7	75.1	116.7	45.5	109.1	45.5
Total cholesterol, mg/dL	229.8	46.6	223.4	38.4	232.8	26.1	216.8	36.8
LDL-cholesterol, mg/dL	145.9	40.5	142.2	33.6	151.1	22.3	130.2 *	27.5
HDL-cholesterol, mg/dL	59.7	11.2	55.7	12.0	56.7	12.7	63.3	23.0

BMI, body mass index; *C. longa*, *Curcuma longa L.*; DBP, diastolic blood pressure; HbA1c, hemo-globin A1c; HDL, high-density lipoprotein; hsCRP, high-sensitivity C-reactive protein; LDL, low-density lipoprotein; SBP, systolic blood pressure. ^1^ Values represent the means and standard deviations for *n* = 19 (placebo group) or *n* = 26 (*C. longa* extract group) in the participants with a low-hsCRP level (<0.098 mg/dL) and *n* = 21 (placebo group) or *n* = 13 (*C. longa* extract group) in the participants with a high-hsCRP level (≥0.098 mg/dL). * *p* < 0.05 vs. placebo group. Results in men and women were compared with the two-tailed Mann Whitney *U*-test, and physical measurements and tests, serum inflammatory markers, and metabolic markers were compared with the two-tailed unpaired Student’s *t*-test when variance was homogeneous or the Aspin-Welch *t*-test when variance was heterogeneous.

**Table 4:** Effect of *C. longa* extract on serum hsCRP and glucose levels stratified by baseline hsCRP levels in the study 1 ^1^.

	Change from Baseline						Repeated Measures Two-Way ANOVA		
	Week 4		Week 8		Week 12		Group	Time	Interaction
	Mean	SD	Mean	SD	Mean	SD			
All study participants									
hsCRP, mg/dL									
Placebo	0.016	0.072	0.036	0.092	0.057	0.138	0.077	0.098	0.243
*C. longa* extract	0.006	0.086	−0.001 *	0.071	0.015 **	0.068			
Glucose, mg/dL									
Placebo	2.2	4.6	2.7	4.7	2.0	5.0	0.070	0.153	0.900
*C. longa* extract	0.1 **	6.5	1.0 *	5.5	−0.1 **	6.6			
Stratified analysis									
Low-hsCRP subgroup (<0.098 mg/dL)									
hsCRP, mg/dL									
Placebo	0.042	0.064	0.055	0.080	0.083	0.150	0.013	0.281	0.387
*C. longa* extract	0.023	0.064	0.020	0.048	0.026 **	0.049			
Glucose, mg/dL									
Placebo	1.2	4.6	2.4	4.7	1.4	5.3	0.576	0.396	0.829
*C. longa* extract	1.0	6.1	1.3	4.5	0.8	5.3			
High-hsCRP subgroup (≥0.098 mg/dL)									
hsCRP, mg/dL									
Placebo	−0.026	0.065	0.007	0.103	0.017	0.111	0.306	0.207	0.233
*C. longa* extract	−0.041	0.124	−0.056 **	0.091	−0.017	0.101			
Glucose, mg/dL									
Placebo	3.6	4.4	3.1	4.8	2.9	4.4	0.052	0.180	0.193
*C. longa* extract	−1.4 **	7.3	0.4 **	7.6	−2.0 **	8.8			

ANOVA, analysis of variance; *C. longa*, *Curcuma longa* L.; hsCRP, high-sensitivity C-reactive protein. ^1^ Values represent the means and standard deviations for *n* = 44 (placebo group) or *n* = 43 (*C. longa* extract group) in all study participants, *n* = 26 (placebo group) or *n* = 29 (*C. longa* extract group) in the participants with a low-hsCRP level (<0.098 mg/dL) and *n* = 18 (placebo group) or *n* = 14 (*C. longa* extract group) in the participants with a high-hsCRP level (≥0.098 mg/dL). * *p* < 0.05 and ** *p* < 0.01 vs. placebo group, as assessed by repeated measures two-way ANOVA followed by a simple test of main effect.

**Table 5:** Effect of *C. longa* extract on serum hsCRP and glucose levels stratified by baseline hsCRP levels in study 2 ^1^.

	Change from Baseline						Repeated Measures Two-Way ANOVA		
	Week 4		Week 8		Week 12		Group	Time	Interaction
	Mean	SD	Mean	SD	Mean	SD			
All study participants									
hsCRP, mg/dL									
Placebo	0.019	0.044	0.036	0.091	0.016	0.047	0.057	0.363	0.302
*C. longa* extract	0.021	0.089	0.002 *	0.032	−0.007	0.035			
Glucose, mg/dL									
Placebo	−2.4	6.3	−1.3	6.3	−0.7	6.1	0.967	0.189	0.433
*C. longa* extract	−1.4	5.5	−1.8	7.2	−0.9	6.3			
Stratified analysis									
Low-hsCRP subgroup (<0.098 mg/dL)									
hsCRP, mg/dL									
Placebo	0.011	0.045	0.039	0.103	0.019	0.050	0.101	0.060	0.831
*C. longa* extract	0.033	0.095	0.010	0.028	0.008	0.022			
Glucose, mg/dL									
Placebo	−3.9	5.5	−1.5	6.0	−1.3	5.6	0.235	0.066	0.193
*C. longa* extract	−0.6 *	5.8	−1.0	6.0	0.4	6.8			
High-hsCRP subgroup (≥0.098 mg/dL)									
hsCRP, mg/dL									
Placebo	0.031	0.041	0.031	0.076	0.010	0.043	0.001	0.334	0.924
*C. longa* extract	−0.024	0.039	−0.019 *	0.035	−0.047 *	0.034			
Glucose, mg/dL									
Placebo	−1.0	6.8	−1.0	6.7	−0.1	6.6	0.187	0.949	0.790
*C. longa* extract	−3.2	4.8	−3.4	9.2	−3.6 *	4.3			

ANOVA, analysis of variance; *C. longa*, *Curcuma longa* L.; hsCRP, high-sensitivity C-reactive protein. ^1^ Values represent the means and standard deviations for *n* = 40 (placebo group) or *n* = 39 (*C. longa* extract group) in all study participants, *n* = 19 (placebo group) or *n* = 26 (*C. longa* extract group) in the participants with a low-hsCRP level (<0.098 mg/dL) and *n* = 21 (placebo group) or *n* = 13 (*C. longa* extract group) in the participants with a high-hsCRP level (≥0.098 mg/dL). * *p* < 0.05 vs. placebo group, as assessed by repeated measures two-way ANOVA followed by a simple test of main effect.

## Data Availability

Data sharing not applicable.

## Supplementary Materials

The following supporting information can be downloaded at: https://www.mdpi.com/article/10.3390/nu14183763/s1. Table S1: Baseline characteristics of the participants in study 1. Table S2: Baseline characteristics of the participants in study 2.
