# Supplementary material for: A Hot Water Extract of Curcuma longa L. Improves Fasting Serum Glucose Levels in Participants with Low-Grade Inflammation: Reanalysis of Data from Two Randomized, Double-Blind, Placebo-Controlled Trials
Source: Nutrients. 2022 Sep 13;14(18):3763. doi: 10.3390/nu14183763 (PMC9500701; doi:10.3390/nu14183763)
Supplement: Supplementary file 1 [file nutrients-14-03763-s001.zip › nutrients-1905761-supplementary.pdf]

Supplementary Table S1. Baseline characteristics of the participants in study 1 [1].

	Placebo (n = 44)		*C. longa* Extract (n = 43)		Total (n = 87)	
	**Mean**	**SD**	**Mean**	**SD**	**Mean**	**SD**
Sex, male/female, n	22/22		23/20		45/42	
Age, y	58.5	5.5	58.8	5.3	58.6	5.4
Physical measurements and tests						
Height, cm	162.1	8.1	162.8	8.3	162.4	8.2
Body weight, kg	66.4	9.4	66.5	7.8	66.4	8.6
BMI, kg/m^2	25.1	2.5	25.0	1.8	25.1	2.2
SBP, mmHg	131.0	16.8	129.2	15.3	130.1	16.1
DBP, mmHg	82.4	11.6	81.6	10.5	81.9	11.0
Serum inflammatory markers						
hsCRP, mg/dL	0.105	0.081	0.090	0.064	0.098	0.073
Metabolic markers						
Glucose, mg/dL	84.2	6.0	86.0	6.9	85.1	6.5
HbA1c, %	5.48	0.24	5.51	0.28	5.49	0.26
Triglyceride, mg/dL	112.5	56.2	127.5	74.1	120.3	66.2
Total cholesterol, mg/dL	220.1	38.6	218.3	33.5	219.0	36.1
LDL-cholesterol, mg/dL	138.1	34.2	132.8	29.9	135.1	32.0
HDL-cholesterol, mg/dL	54.3	14.9	56.0	12.3	55.1	13.6

BMI, body mass index; *C. longa*, *Curcuma longa* L.; DBP, diastolic blood pressure; HbA1c, hemoglobin A1c; HDL, high-density lipoprotein, hsCRP, high-sensitivity C-reactive protein; LDL, low-density lipoprotein; SBP, systolic blood pressure. [1] Values represent the means and standard deviations for n = 44 (placebo group) or n = 43 (*C. longa* extract group) in study 1. Results in men and women were compared with the two-tailed Mann Whitney *U* test, and physical measurements and tests, serum inflammatory markers, and metabolic markers were compared with the two-tailed unpaired Student's *t* test when variance was homogeneous or the Aspin-Welch *t* test when variance was heterogeneous.

Supplementary Table S2. Baseline characteristics of the participants in study 2 [1].

	Placebo (n = 40)		*C. longa* Extract (n = 39)		Total (n = 79)	
	**Mean**	**SD**	**Mean**	**SD**	**Mean**	**SD**
Sex, male/female, n	16/24		15/24		31/48	
Age, y	56.6	4.3	56.7	5.0	56.7	4.6
Physical measurements and tests						
Height, cm	162.5	8.5	162.5	8.5	162.5	8.4
Body weight, kg	69.9	8.0	70.3	7.8	70.1	7.8
BMI, kg/m^2	26.4	1.8	26.6	1.5	26.5	1.6
SBP, mmHg	122.9	12.8	119.7	14.7	121.3	13.8
DBP, mmHg	77.6	10.2	77.6	9.6	77.6	9.8
Serum inflammatory markers						
hsCRP, mg/dL	0.076	0.047	0.075	0.064	0.075	0.056
Metabolic markers						
Glucose, mg/dL	92.3	8.7	88.5*	6.4	90.4	7.9
HbA1c, %	5.63	0.20	5.55	0.26	5.59	0.23
Triglyceride, mg/dL	120.8	52.9	122.2	67.0	121.5	59.9
Total cholesterol, mg/dL	231.4	36.8	221.2	37.5	226.3	37.3
LDL-cholesterol, mg/dL	148.7	31.9	138.2	31.9	143.5	32.1
HDL-cholesterol, mg/dL	58.2	11.9	58.2	16.6	58.2	14.3

BMI, body mass index; C. longa, Curcuma longa L.; DBP, diastolic blood pressure; HbA1c, hemoglobin A1c; HDL, high-density lipoprotein; hsCRP, high-sensitivity C-reactive protein; LDL, low-density lipoprotein; SBP, systolic blood pressure. 1 Values represent the means and standard deviations for n = 40 (placebo group) or n = 39 (C. longa extract group) in study 2. * $p < 0.05$ vs placebo group. Results in men and women were compared with the two-tailed Mann Whitney U test, and physical measurements and tests, serum inflammatory markers, and metabolic markers were compared with the two-tailed unpaired Student's t test when variance was homogeneous or the Aspin-Welch t test when variance was heterogeneous.
